# Elevated glycated hemoglobin levels impair blood pressure in children and adolescents with type 1 diabetes mellitus

**DOI:** 10.1186/s13098-015-0118-0

**Published:** 2016-01-12

**Authors:** Sandra de Oliveira, Dahan da Cunha Nascimento, Ramires Alsamir Tibana, Samuel Lima de Oliveira, Ivo Vieira de Sousa Neto, Roberta Kelly Menezes Maciel Falleiros, Leonardo Garcia Miranda, Hermelinda Cordeiro Pedrosa, James Wilfred Navalta, Guilherme Borges Pereira, Jonato Prestes

**Affiliations:** Department of Physical Education, Graduation Program on Physical Education—Catholic University of Brasilia (UCB), Q.S 7, lote 1—Bloco G—Aguas Claras, Taguatinga, Federal District, Brasilia, DF 71966-700 Brazil; Endocrinology Unity—Research Polo Foundation for Teaching and Research in Health Sciences—Regional Hospital of Taguatinga, Federal District, Brasilia, Brazil; Kinesiology and Nutrition Sciences, University of Nevada, Las Vegas, USA

**Keywords:** Childhood, Cardiovascular health, Diabetes, Glycated hemoglobin, Hypertension

## Abstract

**Background:**

Deregulation of glycemic and glycated hemoglobin (HbA_1_) levels accelerate the progression of cardiovascular complications in type 1 diabetes mellitus (T1DM). The aim of this study was to investigate the association between HbA_1_ and changes in blood pressure of children and adolescents with T1DM.

**Methods:**

A total of 60 children and adolescents were recruited and allocated into two groups (prehypertension and control group). Blood pressure and HbA_1_ were measured by the oscillometric method and high-performance liquid chromatography, respectively.

**Results:**

The prehypertensive group had (*P* < 0.05) higher disease duration, body weight, Z score for body weight, systolic blood pressure (SBP), diastolic blood pressure (DBP) and a higher HbA_1_ when compared with the control children and adolescents. Multiple regression to predict alterations in DBP from HbA_1_ adjusted for age, disease duration, and body mass index demonstrated a positive relationship with DBP (*P* < 0.05). A 1 % increase in HbA_1_ was associated with 1.73 mmHg increase in DBP.

**Conclusions:**

High levels of HbA_1_ may be associated with increased blood pressure in T1DM. A tight control of HbA_1_ levels may provide long-term cardiovascular protection in children and adolescents with T1DM.

## Background

Type 1 diabetes mellitus (T1DM) is associated with several chronic complications, such as hypertension, nephropathy and dyslipidemia. Furthermore, children with T1DM and hypertension present higher pulse pressure and ambulatory stiffness index levels when compared with normotensive patients [1]. Elevated values of blood pressure (BP) in children with T1DM are associated with an increased risk for developing cardiovascular complications later in life [1]. Consequently, the early assessment of hypertension, diagnosis, management and treatment is critical in reducing long term microvascular and macrovascular complications [2]. Currently, children and adolescents with BP levels of 120/80 mmHg or above, but less than 95th percentile, are classified as prehypertensive [3]. Thus, the control of risk factors that might be independently associated with hypertension may improve the care and management of children and adolescents with T1DM [4].

Pietrzak et al. [5], investigated the impact of body mass index (BMI) on BP in 164 patients with T1DM aged 14 years. Results revealed a positive correlation of systolic blood pressure (SBP) and diastolic blood pressure (DBP) with age and BMI, while diabetes duration was only correlated with DBP. Although glycated hemoglobin (HbA_1_) was correlated with BMI, this parameter was not used as a main factor to predict BP elevation in T1DM adolescents, which could be a more sensible indicator as compared with BMI. Interestingly, Rohani et al. [6], evaluated 62 T1DM patients with HbA_1_ < 7 % and observed that 26 % were in the pre-hypertensive stage. Moreover, elevated BP was correlated with the duration of the disease, but not with HbA_1_. Thus, it seems reasonable to investigate the association of different HbA_1_ ranges with BP values in T1DM in children and adolescents.

In a prospective analysis of 9603 middle-aged participants with and without a previous history of diabetes, Bower et al. [7] estimated the association between HbA_1_ and hypertension prevalence. Interestingly, subjects with elevated HbA_1_ had higher mean fasting glucose, BP, BMI, low density lipoprotein (LDL), cholesterol and triglyceride levels. Furthermore, higher baseline HbA_1_ values were associated with increased risk of hypertension during long-term follow-up [7].

Considering that HbA_1_ < 7.5 % is recommended for all pediatric age-groups [4], the lack of correlation between BP and HbA_1_ in the studies from Rohani et al. [6] and Pietrzak et al. [5], may be explained by the well-controlled HbA_1_ of the patients. Thus, a poor HbA_1_ control in T1DM children and adolescents might influence BP.

In this sense, the aim of the present study was to investigate the association between HbA_1_ and changes in BP in T1DM children and adolescents. The initial hypothesis is that the higher HbA_1_ levels the higher BP values.

## Methods

### Study population

A total of 60 children and adolescents with an established diagnosis of T1DM [2] (age: 12 ± 1 years, height: 1.57 ± 0.07 m, weight: 48.58 ± 8.76 kg, body mass index: 19.63 ± 2.96 kg/m^2^, 29 males and 31 females) were recruited from Taguatinga Regional Hospital (Brazil, Brasilia, DF) and participated in the study between February of 2014 and July of 2015.

Participants were classified as prehypertensive (n = 22) or normotensive (n = 38) according to the Fourth Report on the Diagnosis, Evaluation, and Treatment of High Blood pressure in Children and Adolescents [3]. It is now recommended that, as with adults, children and adolescents with blood pressure levels at 120/80 mmHg or above should be considered prehypertensive.

### Anthropometric and body composition evaluation

Height was measured using a stadiometer and weight in a scale, and were used for the calculation of the BMI (kg/m^2^). The Z score for weight, height and BMI were used for analysis and were calculated by growth charts (Centers for Disease Control and Prevention, National Center for Health Statistics, 2000) recommended for clinical and research purposes in children and adolescents. Furthermore, overweight and normal weight groups were separated according to the Z score for BMI according to the World Health Organization (WHO) [8].

### Hemodynamic measurements

Systolic blood pressure, diastolic blood pressure and heart rate (HR) were measured with an oscillometric device (Microlife 3AC1-1, Widnau, Switzerland) according to the recommendations of the Fourth Report on the Diagnosis, Evaluation, and Treatment of High Blood pressure in Children and Adolescents [3]. The cuff size was adapted to the circumference of the arm of each participant according to the manufacture’s recommendations. All office BP measures were assessed in triplicate (measurements separated by 1 min) in a calm environment and with an adequate cuff size, with the mean value used for analysis. During BP measurements participants remained seated quietly in a controlled room temperature environment.

### Blood analysis

Participants reported to the laboratory between 08:00 and 10:00 a.m., after an overnight fast, and after blood collection, samples were centrifuged at room temperature at 2000 rpm for 15 min. All subjects were encouraged to avoid smoking, alcohol and caffeine consumption as well as unusual physical activity to avoid influence on these parameters. All patients provided blood samples and the concentration of glucose (mg/dL), HbA_1_ (%), high density lipoprotein (HDL), LDL, triglycerides and total cholesterol were measured. Glucose and HbA_1_ were measured by high-performance liquid chromatography (Bio-Rad, Brazil). The lipid profile was measured by an enzymatic colorimetric method on autohumalyzer equipment (Human GMBH, Germany). High density lipoprotein was determined by ionic exchange followed by colorimetric reaction with the Linco Research Inc. kit (St Louis, USA).

### Statistical analysis

The results are expressed as mean and standard deviation (SD). The Shapiro–Wilk test was used to analyze the normality of the data. For comparisons between groups, an independent-*t* test was applied and for non-parametric data the Mann–Whitney test was used. A bivariate correlation using Pearson’s test was used to assess the correlation between age, disease duration, HbA_1_ and BMI with BP as the main outcome [9]. The strength and magnitude of the correlation between variables was classified as follows [10]: ±1.0 perfect, ±0.7 to ±0.9 strong, ±0.4 to ±0.6 moderate, ±0.1 to  ±0.3 weak and 0 zero. After that, a multiple regression analysis was performed and BP was analyzed as the dependent variable and adjusted for several confounding variables, such as age, disease duration, and BMI [5]. Significance was accepted at the *P* ≤ 0.05 and analyses were conducted with SPSS version 18.0 (SPSS Inc., Chicago).

The study was approved by the *Fundação de Ensino e Pesquisa em Ciências da Saúde* (nº 710.782) and approved by the Institutional Research Ethics Comitte of Catholic University of Brasilia (nº 681.469) in accordance with the declaration of Helsinki. Informed consent was obtained from parents and informed assent from children and adolescents.

## Results

**Fig. 1:**
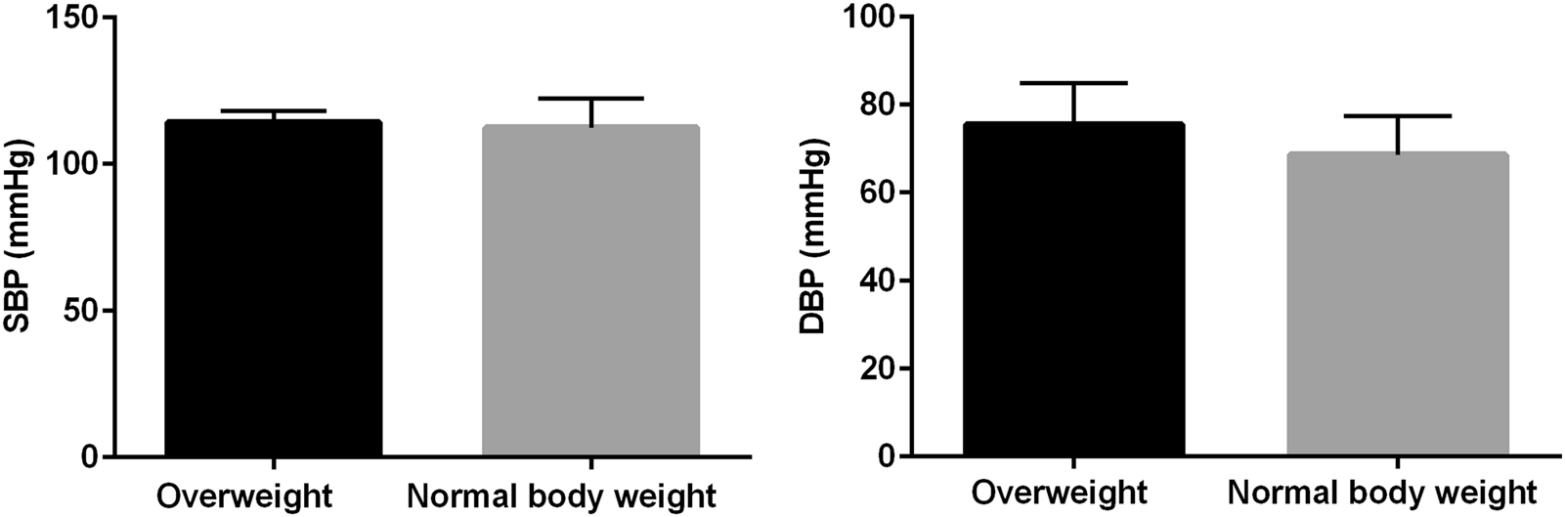
Systolic blood pressure (SBP) and diastolic blood pressure (DBP) between overweight and normal body weight children and adolescents with T1DM. Data presented as means and standard deviation (SD)

Descriptive characteristics of the study participants are shown in Table 1. The prehypertensive group demonstrated significantly higher T1DM disease duration, body weight, Z score body weight, SBP, DBP and HbA_1_ as compared with the normotensive group of children and adolescents with T1DM, although no differences were verified for BMI z-score (Table 1). The analysis according to gender revealed that female individuals displayed a higher basal HR when compared with the male individuals, with no differences for anthropometric and biochemical variables (Table 1). In addition, children’s were divided into overweight and normal body weight. There was no significant difference (*P* = 0.619) in SBP when groups were separated for overweight (114.3 ±1.3 mmHg) and normal body weight (112.4 ± 1.16 mmHg). However, there was a tendency toward statistical significance (*P* = 0.052) for DBP between overweight (75.5 ± 3.33 mmHg) and the normal body weight (68.6 ± 1.4) group (Fig. 1).

**Table 1:** Differences between groups and gender demonstrated by means and standard deviation (SD)

	Normotensive	Prehypertension	*P*	*P* ^b^	Boys	**Girls**
Disease duration, years	5.32 ± 3.62	7.54 ± 3.78*	0.029	0.758	6.31 ± 4.26	6.00 ± 3.38
Age, years	12.78 ± 1.16	13.27 ± 1.20	0.132	0.132	13.20 ± 1.36	12.74 ± 0.99
Body weight, kg	46.40 ± 9.04	52.38 ± 6.90*	0.013	0.852	48.35 ± 8.09	48.80 ± 9.50
Height, cm	1.56 ± 0.06	1.59 ± 0.07	0.082	0.077	1.59 ± 0.07	1.55 ± 0.06
Body mass index, kg/m^2^	19.09 ± 3.10	20.58 ± 2.48	0.080	0.172	19.07 ± 2.30	20.20 ± 3.45
Z score body weight^a^	−0.57 (−4.72 to 1.62)	0.05 (−1.63 to 1.33)*	0.042	0.731	−0.11 (−4.72 to 1.62)	−0.50 (−2.15 to 1.34)
Z score height^a^	−0.73 (−1.89 to 1.68)	−0.21 (−2.21 to 1.18)	0.154	0.747	−0.68 (−1.75 to 1.18)	−0.40 (−2.21 to 1.68)
Z score body mass index^a^	−0.03 (−4.72 to 1.58)	0.23 (−0.95 to 1.17)	0.213	0.642	0.04 (−1.69 to 1.41)	0.15 (−4.72 to 1.58)
SBP, mmHg	107.86 ± 7.32	122.59 ± 6.88*	0.001	0.498	112.34 ± 11.83	114.12 ± 8.22
DBP, mmHg	66.39 ± 5.49	77.04 ± 10.59*	0.001	0.272	68.93 ± 9.83	71.58 ± 8.64
HR, bpm	86.83 ± 15.001	92.27 ± 18.12	0.221	0.024*	83.92 ± 15.22	95.53 ± 16.17
Glycaemia, mg/dl	215.47 ± 90.91	204.00 ± 113.03	0.752	0.978	212.15 ± 82.62	211.21 ± 117.94
HbA_1_, %	9.74 ± 1.98	11.58 ± 3.08*	0.037	0.573	10.17 ± 2.46	10.60 ± 2.67
HDL, mg/dl	54.47 ± 8.45	51.14 ± 12.22	0.333	0.782	52.80 ± 8.48	53.75 ± 12.00
LDL, mg/dl	102.22 ± 25.78	102.50 ± 38.72	0.979	0.814	103.39 ± 30.86	100.93 ± 31.63
Triglycerides, mg/dl	89.44 ± 40.29	92.53 ± 44.39	0.820	0.138	81.91 ± 38.10	101.00 ± 43.57
Total cholesterol, mg/dl	169.78 ± 39.06	177.00 ± 38.67	0.566	0.750	170.52 ± 44.92	174.35 ± 30.87

*SBP* systolic blood pressure, *DBP* diastolic blood pressure, *HR* heart rate, *HbA*_*1*_ glycated hemoglobin

* *P* ≤ 0.05

^**a**^Data not normally distributed is presented as median, minimum and maximum values

^**b**^ Comparisons between sexes

Although not statistically significant, there was a positive weak correlation between age, diagnosis time, HbA_1_ and BMI with blood pressure. In addition, DBP and HbA_1_ demonstrated a positive, significant, and moderate correlation (values of ± 0.5; Table 2).

**Table 2:** Correlation between blood pressure and age, disease duration, HbA_1_ and body mass index

Parameter	Systolic blood pressure	*P*	Diastolic blood pressure	*P*
Disease duration, years	*R* = 0.13	0.297	*R* = 0.00	0.960
Age, years	*R* = 0.12	0.326	*R* = 0.20	0.113
Body mass index, kg/m^2^	*R* = 0.24	0.081	*R* = 0.21	0.113
HbA_1_, %	*R* = 0.24	0.089	*R* = 0.54*	0.001

*HbA*_*1*_ glycated hemoglobin

* *P* ≤ 0.05

Following the correlational analysis, a multiple regression was run to predict alterations on DBP from HbA_1_ adjusted for age, diagnosis time, and BMI (categorical variable used to allocate children and adolescents into eutrophic and overweight according to BMI cutoffs for teenagers and children [8]). The model significantly predicted DBP (CI 95 % for Beta between 0.68 and 2.78, *P* = 0.003), and HbA_1_ demonstrated a positive correlation with DBP. According to this model, each 1 % increase in HbA_1_ is associated with an increase of 1.73 mmHg in DBP (Table 3). For SBP the regression, the adjusted model for the same covariates used in DBP was assessed, and the model did not improve the ability to predict the outcome variable (*P* = 0.11).

**Table 3:** Correlation between HbA_1_ and diastolic blood pressure adjusted for age, disease duration, and body mass index

Variable	*B*	*SE*_*B*_	*β*	*P*
Intercept	29.11	14.80		0.058
HbA_1_, %^a^	1.73	0.51	0.48	0.002*

^a^Adjusted for age, disease duration and body mass index

*HbA*_*1*_ glycated hemoglobin

* *P* ≤ 0.05

## Discussion

There are relatively few studies investigating the impact of HbA_1_ on BP in children and adolescents with T1DM. A multiple regression analysis with adjustment for age, T1DM disease duration and BMI revealed a positive correlation between DBP and HbA_1_. In addition, this study demonstrated that prehypertensive children and adolescents with T1DM presented increased time of disease duration, as well as higher SBP, DBP and HbA_1_ values.

Moreover, comparisons between gender were made in this study, because it is an important predictor of the alteration in carotid artery intima-media-thickness and there is a relationship between CCA and IMT and established cardiovascular disease risk factors, such as hypertension and the quality of the metabolic glycemic control (estimated by HbA_1_) in children and adolescents with T1DM [11]. In addition, in a previous study, boys with T1DM presented higher fasting glucose, lower intima-media-thickness and a lower body mass index (kg/m^2^) when compared with girls with T1DM [11].

The results of this study revealed that HbA_1_ presented a positive correlation with DBP, even adjusted for several confounding variables, such as age, disease duration, and BMI, while no correlation was found with SBP. These results are relevant because, each 1 % increase in HbA_1_ is associated with an increase of 1.73 mmHg in DBP. This increment, although small, can augment the risk of stroke by 14-17 %, and risk of coronary artery disease by 6-9 %, in the general population [12].

The benefits of better BP and lipid levels control were clearly demonstrated by an observational prospective study of 589 patients with childhood-onset T1DM (<17 years) [13]. Higher values for SBP, DBP and lipid levels were associated with higher relative risks for mortality, coronary artery disease and nephropathy. Furthermore, even a small reduction of 2 mmHg in DBP, for example, can have a great impact in terms of number of cardiovascular events prevented, such as coronary heart disease and stroke [14].

Nevertheless, hyperglycemia (evaluated by HbA_1_) that is intimately related with glycemic control, plays an important role in the development of microvascular complications [15] [13]. The levels of HbA_1_ in children and adolescents with T1DM of the present study were higher than the <7.5 % recommended across all pediatric age-groups. Moreover, there is an association of high HbA_1_ levels with hyperglycemia, dyslipidemia and hyperinsulinemia, which mediates vascular dysfunction, activation of the renin-angiotensin-aldosterone system, sympathetic nervous system, and possibly negative alterations in BP [16]. It appears that a lack of association between HbA_1_ and BP in previous studies [5, 6] might be explained by the well-controlled HbA_1_ levels of children and adolescents with T1DM.

Moreover, when comparing children and adolescents with T1DM to obese children and adolescents without T1DM, BP depends on BMI [5, 17, 18]. When groups were separated according to Z score for BMI [8] [8], mean BP values in the groups of patients classified as overweight were higher, but not statistically significant different when compared with the normal body weight group (SBP = 114.25 ± 3.84 vs. 112.44 ± 9.96; DBP = 75.50 ± 9.42 vs. 68.60 ± 8.76 for overweight and normal body mass, respectively).

In addition, prehypertensive children and adolescents with T1DM displayed a longer disease duration and higher BP. Nonetheless, Wigmann et al. [19], found that the elevation of BP, albuminuria, and renal dysfunction were altered in parallel when comparing groups with increased disease duration in 166 T1DM patients aged 25 years. These results suggest that disease duration of T1DM is an interesting topic of research to pursue in further investigations.

The values of glycemic control in this study are similar to the study from Ngwiri et al. [20], where children and adolescents with T1DM from Sub-Saharam Africa presented a median HbA_1_ of 11 %. An interesting result was that age above 12 years was significantly associated with poor glycemic control. The poor glycemic control reported in this study is far from being acceptable and most of the participants of this study might present a high risk for the precocious development of microvascular complications. Therefore, this data strongly reinforce the importance of counseling children and adolescents with T1DM regarding management, follow-up, and more resources should be provided on care of this disease. We agree that the education and follow-up of children and adolescents with T1DM must be analyzed according to economic status, educational level, social level, and available resources. Another important topic to be considered is the viability of educational methods for professionals and the institutions involved. The programs must be coherent as the feasibility, scope, access, cost-effective and proposed goals, and should be adapted according to the health system [21].

It should be noted that this study presents some limitations. Diabetes education differs from country to country, and the control of educational strategies by parents and their children regarding HbA_1_ values that help to prevent long term micro- and macrovascular complications were not controlled in this study. We are aware that education variables in the location where the present study was conducted could be more personalized. However, proper diabetes education for children and adolescents with T1DM, and their family is a complex condition, which requires educators with a set of skills including good communication, compassion, sensitivity, humor, and in-depth knowledge of childhood diabetes [2]. Furthermore, the lack of controlled maturational status [22, 23], daily dose of insulin prescribed, renal status, and physical activity [24, 25] might be important confounding factors that should be analyzed in future studies.

## Conclusions

A poor glycemic control might represent an important factor to be controlled by educational programs as a strategy to prevent hypertension and associated long-term cardiovascular problems in children and adolescents with T1DM. Health behaviors, such as appropriate nutrition and exercise should be recommended as an adjunct to the prescribed medication therapy.

## Abbreviations

HbA_1_: glycated hemoglobin
T1DM: type 1 diabetes mellitus
SBP: systolic blood pressure
DBP: diastolic blood pressure
BMI: body mass index
BP: blood pressure
LDL: low density lipoprotein
HR: heart rate
HDL: high density lipoprotein
SD: standard deviation
